# Non-Canonical Host Intracellular Niche Links to New Antimicrobial Resistance Mechanism

**DOI:** 10.3390/pathogens11020220

**Published:** 2022-02-08

**Authors:** Michaela Kember, Shannen Grandy, Renee Raudonis, Zhenyu Cheng

**Affiliations:** Department of Microbiology and Immunology, Faculty of Medicine, Dalhousie University, Halifax, NS B3H 4R2, Canada; michaela.kember@dal.ca (M.K.); sgrandy@dal.ca (S.G.)

**Keywords:** non-canonical intracellular pathogen, antibiotic resistance, *Pseudomonas aeruginosa*, *Staphylococcus aureus*

## Abstract

Globally, infectious diseases are one of the leading causes of death among people of all ages. The development of antimicrobials to treat infectious diseases has been one of the most significant advances in medical history. Alarmingly, antimicrobial resistance is a widespread phenomenon that will, without intervention, make currently treatable infections once again deadly. In an era of widespread antimicrobial resistance, there is a constant and pressing need to develop new antibacterial drugs. Unraveling the underlying resistance mechanisms is critical to fight this crisis. In this review, we summarize some emerging evidence of the non-canonical intracellular life cycle of two priority antimicrobial-resistant bacterial pathogens: *Pseudomonas aeruginosa* and *Staphylococcus aureus*. The bacterial factors that modulate this unique intracellular niche and its implications in contributing to resistance are discussed. We then briefly discuss some recent research that focused on the promises of boosting host immunity as a combination therapy with antimicrobials to eradicate these two particular pathogens. Finally, we summarize the importance of various strategies, including surveillance and vaccines, in mitigating the impacts of antimicrobial resistance in general.

## 1. Introduction

Infectious diseases remain one of the most pressing challenges faced by humanity today. The recently emerged severe acute respiratory syndrome coronavirus-2 (SARS–CoV-2), the causative agent of the ongoing pneumonia-like pandemic of coronavirus disease 2019 (COVID-19), has infected over 355 million and killed over 5.6 million people worldwide as of January 2022 [1]. This toll on the healthcare system, along with the resultant social and economic turbulences, highlights the importance of pandemic preparedness. Although public health experts believe that respiratory-borne RNA viruses, such as the corona and influenza viruses, are most likely to cause naturally occurring global infectious catastrophes [2], the rapid emergence of bacterial and fungal infections associated with antimicrobial resistance (AMR) also sound the alarm for widespread deadly infections caused by these microorganisms and remain an ongoing priority for risk preparedness [3,4,5,6,7,8]. 

The World Health Organization (WHO) declared AMR as one of the top 10 global public health threats facing humanity in 2019 [9]. Although antimicrobials broadly include antibiotics, antivirals, antifungals, and antiparasitics, the focus of our current review is on antibiotics. The AMR infections pose serious threats to public health, including high mortality rates, unaffordability by the necessity of more intensive care and use of costly alternative drugs, and more risks during surgeries and cancer therapies [10,11,12,13,14,15,16]. The most prominent projection of the severity of the AMR problem was made by Lord Jim O’Neill and their team in the AMR Review entitled “*Antimicrobial Resistance: Tackling a crisis for the health and wealth of nations*”, which predicted that by 2050 there could be about ten million deaths per year due to AMR [17]. This review has been the cornerstone of global actions on AMR control and widely accepted. A recent study published online in January 2022, which presented the most comprehensive investigation of AMR burden to date, estimated there were about 5 million deaths associated with bacterial AMR in 2019 [18]. The development of antibiotics as antibacterial therapeutics revolutionized modern medicine. Paul Ehrlich’s screening and identification of drugs against syphilis [19], together with the discovery of penicillin by Alexander Fleming [20] and its mass production enabled by Howard Florey and Ernest Chain’s purification protocol [21], marked landmark discoveries in modern antibiotics [22]. The broad use of penicillin was followed by the golden era of antibiotics discovery in the period between the 1950s and 1970s. However, no new classes of antibiotics have been discovered in decades. The lack of new treatment combined with the rapid development and spreading of resistance sound the alarm for the AMR crisis. It is crucial to better understand the underlying AMR mechanisms to tackle this problem. Major mechanisms of resistance include: (1) limiting uptake with restricted membrane permeability or by efflux pump; (2) inactivation of drugs; or (3) modification of drug targets [23]. 

In addition, a group of bacteria known as intracellular bacteria can gain entry to host cells, followed by bacterial manipulations of host cell biology to create a unique niche within the host cell environment as part of their infection cycles [24]. Classical examples of intracellular bacteria include members from genus of *Mycobacterium*, *Salmonella*, *Chlamydia*, *Listeria*, *Shigella*, *Legionella*, *Brucella*, and *Rickettsia*. Intracellular bacteria possess unique resistance strategies [25]. To treat intracellular bacteria, different classes of antibiotics can pass host cell plasma membrane via various routes, as β lactams, macrolides, and quinolones pass by diffusion [26], whereas aminoglycosides by receptor megalin-mediated endocytosis [27] (Table 1). However, it is still challenging for antibiotics to reach and stay active inside host cell and sometimes subcellular membrane-concealed organelles where intracellular bacteria hide. Moreover, to survive stresses within the host environment, intracellular bacteria often trigger a physiological switch to a non-replicating or slowly replicating state [28], which also reduces their susceptibility to antibiotics [29]. *Mycobacterium tuberculosis* exemplifies this sophisticated strategy [30]: this bacterium initiates infections by infiltrating the epithelial lining and replicating in alveolar macrophages with bacteria-obstructed phagosome–lysosome fusion. *Mycobacteria* eventually establish latent infections in granulomas, clusters of infected macrophages, neutrophils, epithelioid cells, and foam cells that are encircled by the recruited T and B lymphocytes [31,32]. The non-replicating latent infection state is resistant to conventional treatment [33]. 

*Staphylococcus aureus* and *Pseudomonas aeruginosa*, two priority antimicrobial-resistant bacterial pathogens, are typically considered not to be intracellular. However, emerging evidence suggests that these two species can also be associated with life cycles inside host cells, and while there is an ever-growing mountain of evidence for *S. aureus* as an intracellular pathogen, there is only a handful of evidence of *P. aeruginosa*’s lifestyle as an intracellular pathogen currently available. In this review, we summarize the specific virulence mechanisms underlying this unique intracellular niche and discuss their implications in contributing to resistance in these two priority pathogens. At last, we focus on the discussions about the potentials of combating these two specific pathogens by harnessing host immunity as a combination therapy with antimicrobials and provide a general summary of the key roles of surveillance and vaccines in limiting AMR.

## 2. *P. aeruginosa* as an Intracellular Pathogen 

### 2.1. Overview

*Pseudomonas aeruginosa* is a Gram-negative ubiquitous bacterium and an opportunistic pathogen that is responsible for a wide variety of infections [34]. Typical infections caused by *P. aeruginosa* consist of bacteremia in burn patients, hospital acquired pneumonia in ventilated patients, and urinary tract infections in patients with catheters [34]. *P. aeruginosa* infection is of particular concern to those who are immunocompromised, especially cystic fibrosis (CF) patients [35]. 

Multiple virulence factors contribute to the ability of *P. aeruginosa* to establish both acute and chronic infections. These virulence factors include but are not limited to phospholipase C, proteases, exotoxins, cytotoxins, flagella, pili, and secretion systems [36]. Protein secretion systems are important for delivering virulence factors such as exotoxins and cytotoxins to the extracellular environment and directly into other cells. *P. aeruginosa* utilizes five secretion systems: type 1, type 2, type 3, type 5, and type 6 [37]. The type 3 secretion system (T3SS) and type 6 secretion system (T6SS) allow *P. aeruginosa* to deliver toxins directly into mammalian and bacterial target cells [37]. Both the T3SS and T6SS are implicated in *P. aeruginosa*’s intracellular life cycle as will be discussed below.

*P. aeruginosa* infections are difficult to treat and are becoming an increasing threat due to multiple antibiotic resistance mechanisms including intrinsic, acquired, and adaptive mechanisms. Intrinsic antimicrobial resistance features of *P. aeruginosa* include efflux pumps, low outer membrane permeability, and the synthesis of enzymes that are antibiotic inhibiting/inactivating [38]. Acquired resistance can occur by the acquisition of genes from other species or organisms through the uptake of plasmids, bacteriophages, or transposons [39]. The ability of *P. aeruginosa* to produce biofilms plays a major role in infection and contributes to adaptive resistance; this form of resistance is particularly difficult to treat in infections [40]. 

Another aspect of *P. aeruginosa* that makes it difficult to treat is its striking strain-to-strain diversity. While the average genome size of *P. aeruginosa* ranges from 5.5 to 7 Mbp, the core genome consists of only 321 genes [41,42]. The high plasticity of the *P. aeruginosa* genome contributes to its ability to evade antibiotics and cause a wide range of infections [43]. While clinical isolates tend to have smaller genomes compared with environmental and industrial strains, the antibiotic susceptibility and virulence factors between isolates can vary drastically [43,44]. Therefore, it is difficult to develop a single targeted treatment for all *P. aeruginosa* infections.

While *P. aeruginosa* is traditionally known to be an extracellular pathogen, there is increasing evidence that shows it can invade and survive within host cells. The ability to switch between being an extracellular and intracellular pathogen has been reported for other bacterial species and is a commonly used tactic to evade host defenses [45]. For example, uropathogenic *E. coli* can become an intracellular pathogen, allowing it to establish an intracellular reservoir in bladder epithelial cells, which makes eradication challenging [45]. Since *P. aeruginosa* infections are already difficult to eradicate, the ability of *P. aeruginosa* to establish intracellular reservoirs poses additional complications for treatment. Therefore, understanding how *P. aeruginosa* invades, survives, and persists within host cells is crucial for eradication of infections. 

### 2.2. Intracellular Lifestyle of P. aeruginosa 

Within host cells, *P. aeruginosa* has been shown to aggregate in blebs or biofilm-like structures, alter host cytoskeletal elements, and be tolerant to antibiotics [46,47,48,49]. *P. aeruginosa* has also been found intracellularly in multiple epithelial cell types including lung, corneal, and bladder, as well as phagocytic cells such as macrophages [46,49,50,51]. The strategies employed by *P. aeruginosa* to function as an intracellular pathogen are not well understood; however, the following sections will highlight some of the preliminary discoveries around potential intracellular invasion and survival mechanisms. 

#### 2.2.1. *P. aeruginosa* Invasion of Host Cells 

**Figure 1:**
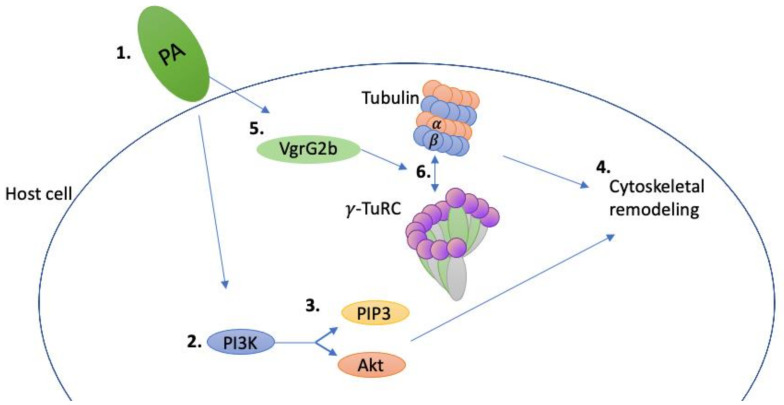
Depiction of *P. aeruginosa*’s ability to subvert the host cell cytoskeleton. When *P. aeruginosa* (PA) binds to epithelial cells (**1**), it results in the activation of PI3K (**2**) in an H2-T6SS-dependent manner [48]. PI3K subsequently activates Akt and PIP3 via phosphorylation (**3**), leading to cytoskeleton remodeling (**4**) [49,52]. Evidence has also shown that the H2-T6SS effector VgrG2b is injected into the host cell prior to *P. aeruginosa* invasion (**5**) [54]. VgrG2b is then able to interact with α/β–tubulin as well as the γ-TuRC (**6**) [45]. This interaction is likely to lead to cytoskeletal remodeling; however, connections between the PI3K and VgrG2b remain to be elucidated.

*P. aeruginosa* possesses three type 6 secretion systems (T6SS) located on three different loci—they are named H1, H2, and H3, respectively [37]. The T6SS allows *P. aeruginosa* to deliver toxins to both prokaryotic and eukaryotic target cells [37]. The H2-T6SS plays a role in the internalization of *P. aeruginosa* into eukaryotic cells; H2-T6SS mutants showed a 75% decrease in *P. aeruginosa* internalization into lung epithelial cells [49]. The T6SS structure resembles the contractile tail of a bacteriophage, allowing it to puncture the target cells to deliver toxins [45]. The H2-T6SS puncturing device contains valine glycine repeat G (VgrG) proteins; a subclass of these proteins called evolved VgrGs, are effectors of the T6SS, and have intracellular functions that involve interacting with the host cells’ actin cytoskeleton [45]. *P. aeruginosa* prefers to enter injured and damaged cells from the basolateral surface and can subvert the cytoskeleton remodeling pathways to invade cells from the apical surface [52]. When *P. aeruginosa* binds to polarized epithelial cells, phosphatidylinositol 3-kinase (PI3K) is activated, resulting in the activation of protein kinase B/Akt (Akt) and the synthesis of phosphatidylinositol 1,4,5-triphosphate (PIP3) [52,53]. Sana and colleagues found that *P. aeruginosa*’s ability to activate PI3K signaling and subsequently subvert cytoskeleton remodeling pathways was dependent on the H2-T6SS [49] (Figure 1). An H2-T6SS mutant (PAO1Δ*clpV2*) cocultured with lung epithelial cells resulted in decreased *P. aeruginosa* invasion and phosphorylation of Akt [49].

Activation of PI3K signaling molecules and their subsequent pathways is required for the internalization of *P. aeruginosa* into epithelial cells because it results in the remodeling of the apical surface into the basolateral surface and protrusions from the cell that consist of PIP3 and actin [52,53]. Later, Sana and colleagues reported that the H2-T6SS effector VgrG2b interacts with the γ-tubulin ring complex (γ-TuRC) as well as the alpha and beta tubulin subunits of microtubules in target cells [45] (Figure 1). VgrG2b is both part of the H2-T6SS structure and a secreted effector, allowing it to enter the host/target cells before the internalization of *P. aeruginosa* [54]. The role of the γ-TuRC is microtubule nucleation, allowing γ-TuRC to have control over when and where microtubule growth occurs [55]. Since Sana and colleagues found that both microtubules and actin are required for the internalization of *P. aeruginosa*, it is likely that it is the interaction between VgrG2b and the γ-TuRC that allows *P. aeruginosa* to subvert the cytoskeleton remodeling pathways allowing for internalization [49]. However, the relationship between VgrG2b, PI3K, and γ-TuRC needs to be further investigated. 

#### 2.2.2. *P. aeruginosa* Intracellular Survival Strategies

The T3SS has been implicated in the intracellular survival of *P. aeruginosa* in phagocytic and non-phagocytic cells. Like the T6SS, the T3SS can deliver its effector proteins across membranes into target cells [37]. Four T3SS effector proteins have been found in *P. aeruginosa*, although no strains encode all four [56]. ExoY and ExoT are encoded by most strains and respectively encode a nucleotidyl cyclase and an enzyme that has both Rho-GTPase activating protein and ADP-ribosyltransferase domains [57,58]. ExoS is the same type of bifunctional enzyme as ExoT, and its expression is typically mutually exclusive with ExoU, a phospholipase [59]. ExoS and ExoT also play an antiphagocytic role that has been shown to prevent uptake into some cell types such as HeLa [60]. The strain-to-strain variability in *P. aeruginosa*’s expression of T3SS effectors results in some strains being cytotoxic through the expression of ExoU rather than ExoS [61]. The cytotoxicity of ExoU-expressing strains prevents them from being invasive, while strains that lack ExoU and express ExoS can be invasive; Table 2 provides examples of ExoU and ExoS expressing strains of *P. aeruginosa* [62]. 

When mutations are made in the needle of *P. aeruginosa* PAO1 T3SS, increased invasion of epithelial cells is observed; however, intracellular survival decreases [47], suggesting that the T3SS plays a role in the intracellular survival of *P. aeruginosa* rather than internalization. Further supporting the role of T3SS in intracellular survival, expression of T3SS increases between 4 and 7 h post infection in intracellular PAO1, suggesting an increasing demand for T3SS [60]. Angus and colleagues observed that wildtype PAO1 formed membrane blebs within corneal epithelial cells that allowed the bacteria to replicate, whereas various T3SS mutants were unable to achieve this [47]. Despite the antiphagocytic properties of ExoS, it is shown to be required for the development of these intracellular bleb niches that *P. aeruginosa* develops [50,60]. ExoS has also been shown to suppress vacuolar acidification, a host mechanism for killing *P. aeruginosa*, thereby contributing to intracellular replication of *P. aeruginosa* [63]. Together these data provide insightful details into the strategies employed by *P. aeruginosa* to survive in the intracellular environment of non-phagocytic cells. 

The T3SS effector ExoS is also important for survival of *P. aeruginosa* within macrophages, as it facilitates escape from phagosomes and macrophage lysis [51]. Cyclic di-GMP is a repressor of T3SS genes, whose expression levels are negatively correlated [51]. Recently, *mgtC* and *oprF* were both identified as promoting intracellular survival within macrophages [51]. When *mgtC* or *oprF* is knocked out in *P. aeruginosa* strain PAO1, higher levels of cyclic di-GMP were detected [51]. As well, *mgtC* and *oprF* mutants were unable to survive intracellularly or induce macrophage lysis [51,64]. Garai and colleagues made T3SS mutant strains and observed that ExoS mutants displayed the same lack of ability to induce macrophage lysis as the *mgtC* and *oprF* mutants, indicating that the T3SS effector also plays a role in inducing cell lysis in macrophages and might be under the control of MgtC and OprF [51]. 

Thus, ExoS of the T3SS promotes survival of *P. aeruginosa* within epithelial cells, allowing the bacteria to evade the host immune response and establish an intracellular niche. In addition to niche establishment, ExoS also contributes to *P. aeruginosa*’s ability to destroy macrophages by allowing it to escape vacuoles and ultimately induce lysis. Therefore, the ability of *P. aeruginosa* to become an intracellular pathogen not only allows it to evade the immune response and antimicrobial therapy, but it also depletes phagocytic cells through ExoS-induced cell lysis. 

Factors other than protein secretion systems have also been found to play an important role in *P. aeruginosa* invasion and survival in epithelial cells. AlgR is a transcriptional response regulator that controls more than 200 genes in *P. aeruginosa* and is required for invasion and intracellular survival of PAO1 in bladder epithelial cells [46]. Phosphorylated AlgR is required for invasion into bladder epithelial cells, while unphosphorylated AlgR is required for intracellular survival of *P. aeruginosa* [46]. Interestingly, lower levels of NF-kB activation were observed in epithelial cells invaded by AlgR mutants [46]. The multi-subunit IkB kinase (IKK) complex is an upstream kinase in the NF-kB signaling pathway that ultimately is responsible for activating NF-kB signaling, and PI3K has also been shown to be able to activate NF-kB [46,65]. When IKK or PI3K are inhibited and cells are infected with *P. aeruginosa*, intracellular survival of *P. aeruginosa* is decreased [46]. However, exogenous activation of the NF-kB pathways through TNFα did not increase the intracellular survival of *P. aeruginosa* [46]. These experiments indicate that while NF-kB signaling is required for maximal survival of *P. aeruginosa* in the intracellular environment of epithelial cells, NF-kB signaling alone is not sufficient to promote survival [46]. NF-kB is required for the clearance of extracellular pathogens including *P. aeruginosa* [46]; thus, the relationship between NF-kB signaling and intracellular survival of *P. aeruginosa* remains to be elucidated. 

### 2.3. Development of Intracellular P. aeruginosa Antibiotic Persister Cells

As mentioned above, biofilm formation plays a critical role in *P. aeruginosa*’s ability to cause acute and chronic infections in the extracellular environment. *P. aeruginosa* can cause membrane blebbing in host cells and establish an intracellular niche that allows for bacterial replication [50]. It has also been observed that *P. aeruginosa* is able to form pods with biofilm-like structures in bladder epithelial cells [48]. These bacterial cells were able to persist in the biofilm-like pods for over 72 h, demonstrating that *P. aeruginosa* can persist for prolonged periods of time in the intracellular environment [48]. Heterogeneity is a classic characteristic of biofilms [66]. Heterogenous gene expression was observed in *P. aeruginosa* cells within the intracellular pods, which is in line with what is typically observed in biofilms [48]. While this is not definitive evidence of biofilm formation, it provides supporting evidence that the pods were intracellular biofilms. Biofilm formation in *P. aeruginosa* is also associated with the development of persister cells. The persister phenotype is enhanced when cells are exposed to stress (e.g., antibiotic treatment or nutrient deprivation), but when the stress dissipates, the wildtype phenotype is restored [67]. Importantly, persisters vary phenotypically from the wildtype but are not the result of a permanent genetic mutation [67]. The ability to revert to the wildtype as well as multidrug tolerance are two hallmarks of persister cells [64]. Within biofilms, some cells experience nutrient limitation, which can induce the persister phenotype, resulting in tolerance to multiple antibiotics [68]. 

A transient antibiotic tolerant phenotype has been observed in intracellular *P. aeruginosa* [46,48]. Intracellular *P. aeruginosa* cells treated with concentrations of ciprofloxacin 10 to 35 times the minimum inhibitory concentration resulted in a biphasic kill curve, where higher concentrations of the antibiotic resulted in increased numbers of tolerant cells [46]. This biphasic kill curve is typical of bacterial persister experiments, suggesting that *P. aeruginosa* is developing persister cells in the intracellular environment [68]. As well, when the bacteria were isolated from the intracellular environment, the wildtype susceptibility to ciprofloxacin was restored, demonstrating the ability of the tolerant cells to revert to the wildtype [46]. Garcia-Medina and colleagues observed the same reversible antibiotic tolerant phenotype in cells isolated from the intracellular biofilm pods they reported [48], further supporting the idea that *P. aeruginosa* is developing persister cells in the intracellular environment. The idea that an intracellular pathogen could develop persister cells within the host is not unheard of: *Salmonella typhimurium* cells within macrophage vacuoles adopted a non-replicative state typical of persister cells, allowing it to achieve long term survival against host damage and antibiotics [69,70]. 

### 2.4. Summary

There are multiple factors that allow for intracellular invasion and promote survival of *P. aeruginosa* in the intracellular environment. However, information around specific mechanisms, such as how NF-kB signaling provides protection or how ExoS induces membrane blebbing, is still unknown. Even more questions arise around mechanisms underlying the development of intracellular biofilms and persister cells. What is clear is that *P. aeruginosa* possesses the ability to not only invade host cells but to persist within epithelial cells for extended periods of time, allowing for a potential reservoir of bacteria, contributing to the already difficult task of eradicating *P. aeruginosa* infection. 

## 3. *S. aureus* as an Intracellular Pathogen

### 3.1. Overview

*Staphylococcus aureus* is a Gram-positive commensal bacterial species that is well-recognized as a major human pathogen [71]. Roughly 30% of humans are colonized with *S. aureus*, with the human nose acting as the predominant reservoir [72]. *S. aureus* is responsible for a wide spectrum of infections ranging from various soft tissue infections to invasive systemic infections [73]. As of 2021, it remains the dominant Gram-positive bacteria responsible for aggressive nosocomial infections [74]. Despite its notorious profile as an aggressive extracellular organism, *S. aureus* is additionally recognized as a facultative intracellular pathogen: it is capable of entering host cells, replicating, and persisting in the host cells as part of its survival strategy [75,76,77]. The potential benefits of this practice are twofold in nature: intracellular persistence helps *S. aureus* avoid detection by professional phagocytes, evading host bactericidal defenses and detection by host pattern recognition receptors; it additionally offers concurrent protection from antibiotic therapies. Such factors impact patient recovery from staphylococcal infection while concomitantly increasing the severity of illness [78]. Intracellular localization of *S. aureus* has been suggested to contribute to the often prolonged, chronic course and frequent relapse of severe infections, such as endocarditis and osteomyelitis [79,80,81].

### 3.2. Intracellular Lifestyle of S. aureus 

#### 3.2.1. Internalization and Cell Entry

**Figure 2:**
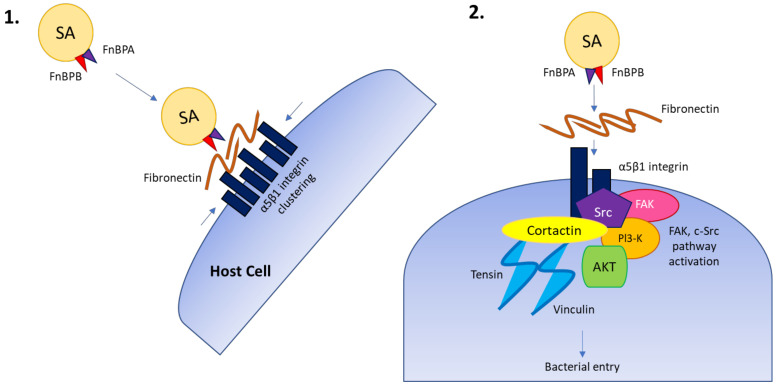
Overview of the zipper-type mechanism or FnBP-Fn-α5β1 integrin-mediated uptake, involving staphylococcal fibronectin-binding proteins A and B (FnBPA and FnBPB). *S. aureus* (SA) contains fibronectin-binding proteins A and B (FnBPA and FnBPB) (**1**). As described by Liang and colleagues [85], these proteins bind to host α5β1 integrin molecules on the surface of cells (**1**) where FnBPA repeats bind to Fn and encourage the clustering of α5β1 integrins. The clustering of integrins promotes the recruitment of host proteins (**2**), including vinculin and tensin, and will additionally promote activation of host focal adhesion kinases (FAKs) and proto-oncogene tyrosine-protein kinase Src (Src) to the bacterial attachment site. The combined activity of FAK and Src results in tyrosine phosphorylation of several host effectors that trigger cytoskeletal rearrangements and the assembly of characteristic endocytic complexes on the intracellular side of the plasma membrane to allow bacterial entry [85].

*S. aureus* invades a broad range of non-professional phagocytic cells (NPPCs) including epithelial/endothelial cells, osteoblasts, fibroblasts, and keratinocytes, amongst others [74,82]. The adhesion and invasion of NPPCs by *S. aureus* is typically achieved by the well-documented zipper-type mechanism, “FnBP-Fn-α5β1 integrin-mediated uptake”, involving staphylococcal fibronectin-binding proteins A and B (FnBPA and FnBPB) [83,84]. Various cellular pathways are triggered during *S. aureus* binding to host cells, contributing to the endocytosis of the bacterium during cellular invasion. In the model described in Figure 2 of the major internalization pathway involving FnBPA, Fn, and the α5β1 integrin described by Liang et al. [85], the dimeric host Fn will bind to α5β1 integrin molecules on the surface of cells. FnBPA repeats bind to Fn and expose α5β1 binding sites that encourage the clustering of α5β1 integrins. Clustering of integrins promotes the recruitment of host proteins, including vinculin and tensin, and will additionally promote activation of host focal adhesion kinases (FAKs) and proto-oncogene tyrosine-protein kinase Src (Src) to the bacterial attachment site. The combined activity of FAK and Src results in tyrosine phosphorylation of several host effectors that trigger cytoskeletal rearrangements and the assembly of characteristic endocytic complexes on the intracellular side of the plasma membrane to allow bacterial entry [85]. Alternative staphylococcal host proteins may support the FnBP-Fn-α5β1 integrin-mediated uptake pathway in certain cell types, including the extracellular adherence protein (Eap), which mediates adhesion and internalization of *S. aureus* into keratinocytes at various stages of development [86]. 

**Figure 3:**
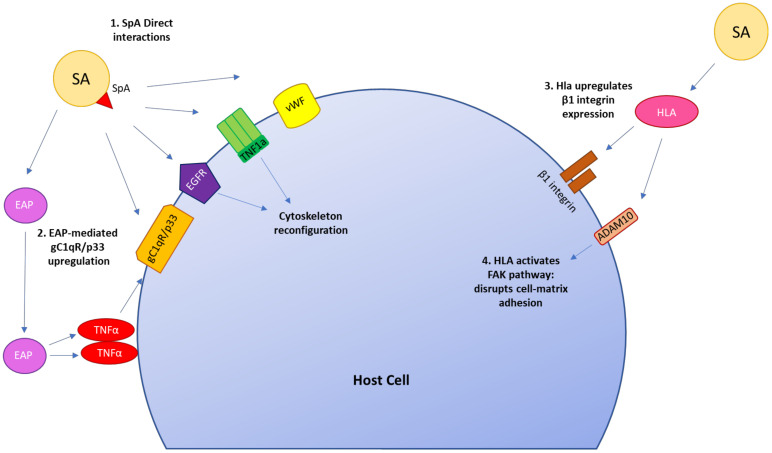
Alternative staphylococcal mechanisms for cellular entry. Staphylococcal protein A (SpA) directly interacts with host tumor necrosis factor α receptor 1 (TNF1a) [106], host receptor gC1qR/p33 on endothelial cells [107], and host vWF in the extracellular matrix of human umbilical vein endothelial cells (**1**) [108]. SpA has been shown to activate TNF1a and EGF receptor (EGFR) signaling cascades that will re-configure the cytoskeleton for staphylococcal internalization [109,110]. Staphylococcal protein EAP may also enhance attachment of SpA to the endothelium by upregulating host receptor gC1qR/p33 on endothelial cells via TNFα release in the bloodstream (**2**) [107]. *S. aureus* (SA) has been additionally shown to stimulate its own uptake by upregulating β1 integrin expression in the host cell through the secretion of α-hemolysin (HLA) (**3**) [111,112]. *S. aureus* HLA will disrupt cell-matrix adhesion by activating FAK signaling via interaction with transmembrane protein ADAM10 with the consequent acceleration of focal contact turnover to overcome the defensive barrier function of the airway epithelium (**4**) [113]. This FAK will also cause plasma membrane depolarization and activates p38 MAP kinase [114]. The β1 integrin is additionally involved in transient activation of the phosphatidylinositol 3-kinase/Akt signaling pathway, which might play a crucial role in β1 integrin-mediated internalization of *S. aureus* [115].

While the FnBP-Fn-α5β1 integrin pathway is historically recognized as the main internalization process, there are numerous “secondary” mechanisms between *S. aureus* virulence factors and host cell components that are involved in or mediate FnBP-independent cellular invasion by *S. aureus*. These mechanisms involve major staphylococcal proteins such as autolysin (Atl), α-hemolysin (HLA), clumping factor A (ClfA), clumping factor B (ClfB), iron-regulated surface determinant B (IsdB), lipoprotein-like lipoproteins (Lpls), serine-rich adhesin for platelets (SraP), and bacterial serine aspartate repeat-containing protein D (SdrD), whose variable interactions with host cell receptors are summarized in Table 3 [87,88,89,90,91,92,93,94,95,96,97,98,99,100,101,102,103,104,105]. Further entry pathways are summarized in Figure 3 [106,107,108,109,110,111,112,113,114,115]. A thorough summary of the various staphylococcal mechanisms involved in cellular adhesion and invasion is reviewed by Josse et al. [116]. 

Many of the *S. aureus* virulence factors that have been shown to act as both primary and secondary entrance mechanisms are unequally distributed throughout *S. aureus* strains and between the core genome and accessory genomes of those strains [117]. Genome-scale models have been created to analyze both the shared and unique virulence factors found between *S. aureus* strains [117]. However, there appears to be a lack of consensus across various genomic investigations regarding which virulence factors are members of the core genome and which are not. For example, as sequencing technology advances, virulence factors that were presumed core genome members have showed greater variation between strains than previously thought. In a study published by Bosi et al. [117] to investigate the conservation of virulence factors across the *S. aureus* strains, the authors selected a set of known virulence factors present in 64 different strains based on literature and database searches. They discovered that multiple virulence factors considered “core genome members” were not found across all 64 strains. Examples of this include protein A (encoded for by *spa*), which was found in 90% of strains, and α-hemolysin (encoded for by *hla*), found in 96% of strains [117]. Similarly, Lebughe et al. [118] discovered that *hla* was detected in 90.3% of the 186 *S. aureus* isolates that were paneled [118]. These variable findings complicate how to determine which virulence factors may be present within the core genome of *S. aureus* [118]. A comprehensive list of the percentage of isolates recognized to contain the genes encoding for the primary and secondary mechanisms utilized for cellular entry by *S. aureus* is summarized in Table 4 [117,118,119,120,121,122,123,124,125], illustrating the broad range of variations that exist across *S. aureus* strains’ genomes.

#### 3.2.2. *S. aureus* Intracellular Survival Strategies

Once endocytosed by host cells, *S. aureus* can remain there for significant periods of time [82,84,126,127]. Indeed, viable bacteria have been recorded inside human endothelial cell lines for up to 10 days after infection and inside THP-1 macrophages up to 7 days [126]. Sinha and Frauholz [84] note that the fates of *S. aureus* and the survival strategies that it employs to persist in the infected host cell depend on a wide variety of factors that aid in intracellular persistence, including staphylococcal isolate and corresponding virulence factors and differential susceptibility of host cells to virulence factors based upon host cell type and respective gene expression [84].

To remain in professional phagocytes, *S. aureus* must defend against the cell’s intracellular antibacterial measures. *S. aureus* has been shown not only to survive but even to replicate inside the harsh phagosomal environment. *S. aureus* variably alters the phagosomal acidification process and its eventual fusion with the lysosome, although such interaction is heavily dependent upon the staphylococcal strain and host cell type. The *S. aureus*-containing endosome acquires early endosomal markers such as the small GTPase Rab5 that transition to a Ras-related protein Rab7a and lysosome-associated membrane protein 1 (LAMP1)-associated phagosome that has an acidic intracellular environment [128,129,130,131]. This acidification plays a variety of roles in the prolonged survival and replication of *S. aureus* within the cell, with some studies reporting different staphylococcal strategies used to cope with acidification. Acidification in macrophages promotes the expression of the accessory gene regulator (agr) system, an intracellular quorum sensing system of *S. aureus* crucial for the upregulation of numerous virulence factors [131]. Furthermore, intracellular *S. aureus* will upregulate multiple peptide resistance factor (MprF) through its GraRS system to begin the process of replication within acidified phagolysosomes [132]. Other staphylococcal factors produced to support the de-acidification of the immediate intracellular environment include urease, arginine deiminase, and nitrite/nitrate reductases [130]. The acidification step may be in some cases crucial for *S. aureus* proliferation: the presence of inhibitors of phagosomal acidification was shown to significantly decrease the intracellular survival rates of *S. aureus* in THP-1 macrophages [131]. However, others report an inhibition of acidification or a complete failure of phago-lysosomal development in *S. aureus*-containing cells [133], as the acidic environment is not achieved due to the lack of production of key host lysosomal hydrolases such as beta-glucuronidase and cathepsin D [131,134]. This may potentially occur via *S. aureus* disruption of beta-glucuronidase and cathepsin D activation [131,134]. Still others have argued that modulation of phagosomal acidification does not affect intracellular survival of *S. aureus* at all [130].

*S. aureus* additionally recruits or produces multiple factors to deal with the host cell’s innate immune response to invasion: genetic factors, including the dlt operon, will encode for proteins responsible for the d-alanylation of teichoic acids in the *S. aureus* membrane. These *S. aureus* proteins provide a physical barrier against host cell defensins and antimicrobial peptides [132]. Multiple peptide resistance factor (MprF) will also confer bacterial resistance against host cationic antimicrobial peptides [132]. Finally, staphyloccocal alternative sigma factor SigB expression contributes to *S. aureus* intracellular persistence by aiding in wildtype *S. aureus* transition to small colony variants (SCVs), which are adapted for long-term intracellular persistence [127]. Other proteins including alpha-toxin, the metalloprotease aureolysin, protein A, and sortase A are also identified as crucial for *S. aureus* persistence in human monocyte-derived macrophages, but their specific roles remain unclear [135].

Extended intracellular persistence of *S. aureus* offers more than a place for replication; in some cases, macrophages may serve as vehicles for the dissemination of infection [135]. *S. aureus* replicates within macrophages during a critical window of time where overexposure to infectious agents has resulted in infected macrophages briefly losing their ability to kill pathogens (termed as “exhausted” macrophages) [134]. *S. aureus* emerges from these cells via host-cell lysis [130,131,134,136]. The emerging *S. aureus* can be phagocytosed again by macrophages to produce a cyclical pattern of uptake and escape that creates a pool of *S. aureus* within macrophages [128,134,136]. The survival of *S. aureus* within exhausted macrophages is hypothesized to cause the dissemination of bacteria throughout the host, potentially resulting in deeper and more aggressive infections [134]. This theory has been separately supported by studies employing the intravenous injection of peritoneal macrophages with *S. aureus* to demonstrate higher levels of bacterial dissemination to the kidneys and brain when compared with infections with planktonic bacteria [137]. Surewaard et al. [138] found that captured *S. aureus* will replicate long-term in liver macrophages, and the eventual release of massive amounts of *S. aureus* from the lysis of dying cells will cause devastating bloodstream infections. Similarly, infected liver neutrophils have been shown to re-enter circulation after infection and disseminate the pathogen to secondary infection sites [139].

#### 3.2.3. *S. aureus* Intracellular Escape and Replication

*S. aureus* phagosomal escape and subsequent cytoplasmic replication is observed in many non-professional phagocytes such as epithelial cells [129,140], endothelial cells [82,129], and keratinocyte lines such as HaCaT or RHEK-1 [82,141]. *S. aureus* phagosomal escape usually relies upon upregulation of the agr system [141,142], and *S. aureus* mutants lacking agr will not translocate to the host cell cytoplasm [82]. Agr activity will produce α-type phenol-soluble modulins (PSMs), including staphylococcal δ-toxin and PSMα1–4, widely considered key mediators of phagosomal escape in *S. aureus* strains [129,143,144]. Despite their role in *S. aureus* translocation, PSMα production alone is not sufficient for the induction of escape: overexpression of PSMα in escape-deficient *S. aureus* strains did not result in bacterial translocation to the host cytoplasm [140].

Other contributing factors to *S. aureus* escape may include the β-type PSMs, as proteomic analysis of *S. aureus* strains capable of phagosomal escape has found that these strains produce both PSMα and PSMβ proteins. Still others additionally upregulate the δ-toxin gene *hld* [143] or express the phospholipase β-toxin prior to escape [140]. The inactivation of PSM-specific smα or ABC-transporter component pmtC reduced phagosomal escape of *S. aureus* [82,145]. The production of pore-forming toxins α-toxin (Hla) and Panton–Valentine leucocidin (PVL) is critical for escape from some cell types and unneeded for others [129,141,143,146,147]. Other staphylococcal proteins such as the Tet38 efflux pump have been shown to be involved in bacterial escape from phagosomes in epithelial cells, but it is still unknown how this interaction mediates escape [143,148]. Additional phagosomal escape factors have been identified, including a nonribosomal peptide synthetase (NRPS) complex AusAB in HeLa cells [140].

### 3.3. S. aureus Influence over Autophagy and Host Cell Death Pathways

#### 3.3.1. Intracellular *S. aureus* Influence over Autophagy

Autophagy is a degradation procedure responsible for the elimination of unwanted cytoplasmic components and is utilized by the cell to degrade invading pathogens into presentable antigens for host defense [149,150,151]. *S. aureus* has been shown to trigger an autophagic response in a wide variety of non-professional phagocytes (NPPCs) as well as dendritic cells, macrophages, and neutrophils [152,153]. *S. aureus* has been shown to evade host autophagy, and *S. aureus* strains that undergo agr upregulation will block the process of autophagy within dendritic cells, leading to the cytotoxic accumulation of autophagosomes [128,154]. Others have reported that such *S. aureus*-containing phagosomes can mature into phagolysosomes without triggering the autophagy pathway [128]. However, some staphylococcal strains were shown to utilize autophagic machinery for survival. Phagocytes will degrade invading microbial threats in LC3-associated phagocytosis (LAP), which combines phagocytic processes with autophagy, resulting in association of microtubule-associated proteins 1A/1B light chain 3 (MAP1LC3) with the phagosomal membrane. The LC3-decorated phagosomes go on to fuse with lysosomes, resulting in enhanced killing and degradation of contained pathogens [155]. The LAP response in macrophages is used as a replication niche for *S. aureus* [152,154,156]. However, even while containing replicating *S. aureus*, these phagosomes still continue to express selective xenophagy receptor p62 on these phagosomes, which is contrastingly associated with host protection [153,156]. Therefore, while the formation of LC3-positive phagosomes facilitates the intracellular replication of *S. aureus*, the retained expression of p62-positive phagosomes represents the host still struggling to eliminate *S. aureus* [156].

Host cell autophagic processes are manipulated by intracellular *S. aureus* in NPPC in multiple ways. However, it remains unclear whether *S. aureus* specifically manipulates autophagy to mitigate host cell defenses or increase its nutrient load or whether it uses autophagic machinery as a replicative niche for increased dissemination. *S. aureus* releases the α-hemolysin (Hla) toxin to trigger the autophagic pathway within NPPC [153,157,158]. However, Hla will activate the host cell inflammasome response [159] through acid sphingomyelinase activity [160], which is detrimental to *S. aureus*. To combat this host-innate immune response, intracellular *S. aureus* develops mutations in the agr regulon (reviewed in Bronesky et al. [161]) that could eliminate production of Hla to decrease inflammasome activation while preserving autophagy. Soong et al. [162] report that *S. aureus*-infected keratinocytes undergoing induced autophagy displayed lower inflammasome activation and reduced mortality, while inhibition of autophagy resulted in increased cell death levels. The subversion of autophagy to impede programmed cell death (PCD) has also been observed in polymorphonuclear leukocytes, wherein *S. aureus* is found surviving in autophagosomes and replicating while simultaneously inhibiting apoptosis [163]. Neumann et al. [153] demonstrated the recruitment of selective autophagy receptor proteins such as p62 by murine fibroblasts in response to intracellular infection with *S. aureus*. In response, *S. aureus* activated a strong anti-autophagic response via upregulation of the MAPK14/p38α MAP kinase-mediated blockade of autophagy, which inhibits fusion with lysosomes to escape degradation [153]. *S. aureus* did not require autophagosomes to further its development and actively inhibited autophagosomal maturation. A study suggesting yet another alternative benefit for autophagy noted that a significant number of autophagosomes did not contain *S. aureus* in infected HeLa cells [164]. Instead, *S. aureus* significantly altered the host metabolic state, simultaneously activating autophagy. By doing so, it began to divert the nutrients generated by this pathway to meet its metabolic needs [154,164]. It was additionally noted in this study that when a reduction in autophagy was observed, *S. aureus* survival decreased, potentially due to loss of nutrients from alteration of host metabolism. While *S. aureus* likely exploits host cell autophagy, it remains unclear whether this is critical for nutrient uptake, self-defense, the creation of a replication niche, or a combination of all three [157,165].

#### 3.3.2. Intracellular *S. aureus* Influence over Host Cell Death

*S. aureus* has been identified as manipulating all known principal mechanisms of PCD, including apoptosis and pro-inflammatory pathways necroptosis or pyroptosis, in NPPCs [166,167,168,169]. There are multiple potential benefits derived from *S. aureus* modulation of host cell death. First, controlling the timing and mode of host cell death may help intracellular *S. aureus* maintain its replicative niche for an extended period of time [168,169]. It can additionally utilize host cell death to penetrate host cell barriers, supporting its dissemination into surrounding tissues [166,167]. Finally, control over host cell death may provide intracellular *S. aureus* with shelter from host immune responses and antibacterial therapies [166].

*S. aureus* has factors that specifically inhibit apoptosis in NPPCs to delay host cell death and control bacterial exit from host cells [170]. Korea et al. [170] suggested that *S. aureus* utilizes EsxA, an effector protein secreted by the staphylococcal type VII secretion system, to inhibit apoptosis. This may potentially give the bacterium time to replicate and disseminate into the host tissues [170]. Contrastingly, studies have shown that apoptosis is induced by *S. aureus* in keratinocytes, as shown by the activation of apoptosis caspases (caspase-3, -7, and -8) [171,172]. The trigger for apoptosis in keratinocytes may be the production of PVL, as Chi et al. [141] demonstrated that a PVL-producing *S. aureus* strain caused significantly more caspase-dependent apoptosis in keratinocytes than a strain lacking PVL production.

*S. aureus* additionally interacts with pyroptosis pathways in NPPCs. Pyroptosis induction has been shown to be a crucial step required for *S. aureus* penetration through keratinocyte barriers, with Soong et al. [162] suggesting that *S. aureus* may activate pyroptosis of keratinocytes to enhance dissemination [162].

*S. aureus* produces a broad range of pro-PCD virulence factors that may facilitate infiltration of the bacteria into the cells and surrounding tissues, as reviewed previously [168,173], to delay host cell death for increased dissemination or to penetrate host cell barriers. Additionally, other studies note that *S. aureus* may attempt to prolong NPPC life and avoid triggering these pathways to ensure safety from antibacterial treatment. For example, Kindi et al. [174] noted that *S. aureus* entered and proliferated within keratinocytes without killing them or triggering any host cell death pathways. The majority of anti-staphylococcal antibiotics at 20-fold their standard minimal inhibitory concentration, including flucloxacillin, teicoplanin, clindamycin, and linezolid, did not kill internalized *S. aureus* during this time. The internalization of *S. aureus* by human skin keratinocytes and subsequent lack of host cell death pathway completion in response allowed *S. aureus* to evade killing by most anti-staphylococcal antibiotics in this study [174].

*S. aureus* may trigger more than one of the above pathways at once: Steltzner et al. [175] suggest that intracellular *S. aureus* disturbs Ca^2+^ homeostasis and induces cytoplasmic Ca^2+^ overload in HeLa cells, which results in both apoptotic and mitochondrial permeability transition-driven necrotic cell death occurring simultaneously (or in quick succession). However, they suggest that the damage induced by strong perturbations of the cellular Ca^2+^ homeostasis and Ca^2+^ overload may not allow reliable detection of specific signaling pathways. With so many potential interactions between *S. aureus* and PCD pathways, it is difficult to target what the specific pathogenic goal of PCD may be; the damage resulting from this Ca^2+^ overload may be the reason for much of the conflicting information [175].

Intracellular *S. aureus* has also been shown to trigger pyroptosis, necroptosis, and other necrotic pathways in THP-1 macrophages, monocytes, and neutrophils, with minimal activation of apoptosis reported in phagosomes of these respective cells [147,176,177,178]. As seen in NPPC, more than one pathway may be simultaneously activated: Flannagan et al. [128] found that *S. aureus*-infected macrophages showed characteristics of both apoptosis and necrosis [128]. Other studies of *S. aureus*-infected neutrophils have shown that the cells will undergo morphological changes consistent with apoptosis, before necroptotic RIPK-1-dependent lysis occurred [179].

Bacterial control over professional phagocytic cell death is thought to support bacterial dissemination throughout the body as they travel within these mobile cells. Koziel et al. [180] suggested that *S. aureus* maintain human monocyte-derived macrophages for extended periods despite the appearance of early apoptotic features in host cells. *S. aureus* strongly upregulated the expression of *B-cell lymphoma 2* (*BCL2*) and *myeloid cell leukemia 1* (*MCL1*) genes, which stabilize mitochondrial membrane potential and delay apoptosis. The protective effects of *S. aureus* were confirmed by the finding that *S. aureus*-infected macrophages were more resistant to staurosporine-induced cell death than control cells [180]. Similarly, another study found that intracellular *S. aureus* infection in dendritic cells delayed host cell apoptosis, implicating staphylococcal protein EsxA as a critical mediating factor during this process [181].

### 3.4. Summary

*S. aureus* has now been recognized as a facultative intracellular pathogen, with its ability to adeptly shift from an extracellular to an intracellular lifestyle playing a crucial role during recurrent and aggressive infections. *S. aureus* enters a wide range of host cell types and can replicate and survive within host cells, utilizing an incredible range of host cell pathways to enable its survival. As a result of this, effectively eliminating intracellular *S. aureus* remains a major challenge.

## 4. Treating Intracellular *P. aeruginosa* and *S. aureus* by Harnessing the Host Immune Response in Combination with Using Antimicrobials

For a treatment to successfully eradicate intracellular bacterial pathogens, the treatment must overcome several physical barriers due to the location of the pathogens. An obvious treatment strategy would be to enhance and manipulate the host’s immune response to the intracellular pathogen, with the most logical treatment choice being a vaccine. Vaccines are one of the most effective strategies for engaging and enhancing the host’s natural immune response to prevent and/or eradicate an infection by a pathogen. However, in the case of *S. aureus* and *P. aeruginosa*, no vaccines are currently available, as discussed below. Additionally, both bacterial species discussed here are listed as species of concern by the WHO due to their AMR, and as discussed above, are both highly adaptable pathogens, common in the environment, and capable of forming biofilms and both have a multitude of virulence factors that can aid in infection, host evasion, and antibiotic resistance/tolerance. Consequently, treating these two pathogens is both challenging and situational, depending on the type or location of the infection and the antibiotic tolerance and resistance profile of the specific strains involved [73,182].

Combination therapies show promise for treating these two non-canonical intracellular pathogens, including therapies that combine traditional antimicrobials with treatments that harness the host’s immunity. While vaccines are an active immune-based therapy, the use of antibodies or antisera are common passive immune-based therapies. There are many antibody-based treatments that target various antigens from *P. aeruginosa* or *S. aureus* in various stages of clinical trials, as reviewed in [183,184,185]. Compared with antibiotic treatments, antibody-based treatments can be expensive due to the amount of protein required per dose, though there are more cost-effective methods available to treat the host with antibodies, such as using DNA-encoded monoclonal antibodies (DMAbs) that are injected into the host’s muscle cells, where the antibodies can be made and then circulated to infections sites. DiGiandomenico et al. [186] designed a DMAb that is bispecific and targets PcrV (a protein required for the type III secretion system activity) and PsI (an abundantly expressed exopolysaccharide) from *P. aeruginosa*. The bispecific DMAb, MEDI3902, showed synergy with the antibiotics ciprofloxacin and tobramycin in mouse models of infections and even at subtherapeutic doses of MEDI3902 with either antibiotic, the combination treatments were effective in preventing death in mice [186]. Unfortunately, MEDI3902 was abandoned in phase II of clinical trials in 2020 [187].

Other antibody-based therapies have shown potential against intracellular *P. aeruginosa* and *S. aureus* infections. An antibody–antibiotic conjugate (AAC) was constructed by Lehar et al. [137] from Genentech Inc. using monoclonal antibodies (MAbs) against β-GlcNAc WTA, β-O-linked N-acetylglucosamine (GlcNAc) sugars on wall-teichoic acids (WTAs), which was linked to the highly effective bactericidal antibiotic rifalogue (a rifampicin derivative) using a cathepsin-cleavable covalent linker. These AACs opsonize *S. aureus*, and once they are phagocytized, the rifalogue is cleaved from the antibody by endoenzymes within the host cells, and the antibiotic effectively kills intracellular *S. aureus*, since rifalogue is effective against viable, replicating, and non-replicating bacteria. In vitro and in vivo testing showed this AAC was highly effective at reducing pathogen loads. Additionally, in a mouse infection model, the AAC was more effective at treating *S. aureus* bacteremia than a traditional treatment with vancomycin antibiotic [137]. Recently, some of these same researchers from Genentech Inc. completed a proof-of-concept study where they engineered an AAC that was effective against *P. aeruginosa* [188]. Building on their previous work, this AAC consisted of MAb 26F8, which binds to lipopolysaccharide O, covalently linked with a lysosomal cathepsin-cleavable linker to the G2637 antibiotic. The AAC, 26F8-cBuCit-G2637, effectively cleared intracellular *P. aeruginosa* inside macrophages. While demonstrating great in vitro efficacy, unfortunately the 26F8-cBuCit-G2637 AAC was not therapeutically effective in a mouse model of acute *P. aeruginosa* pneumonia, and the researchers suggest that one possible solution to this hindrance could be to use a MAb with stronger opsonic activity to improve the effectiveness in vivo [188].

While the above two examples with AAC-based therapies only passively utilize the host in the therapy against intracellular pathogens, there are some promising new studies that utilize repurposed drugs to directly enhance the host’s response to the pathogen in combination with traditional antibiotics. Evans et al. [189] investigated co-treatments of rifampicin with two statin drugs, simvastatin or ML141, against intracellular *S. aureus* infections. While each statin drug acted on the host and reduced invasion of *S. aureus* due to reductions in actin stress fiber reordering and fibronectin binding, only the co-treatment of ML141 and rifampicin decreased intracellular *S. aureus* compared with the rifampicin-alone treatments. Thus, the host-directed therapy of ML141 inhibited bacterial invasion and enhanced rifampicin-killing of *S. aureus*, as this lipophilic antibiotic is effective against intracellular and extracellular bacteria [189]. A different host-directed therapy strategy was utilized by Xiao et al. [190], as they used the drug memantine (MEM), which is an FDA-approved drug to treat moderate to severe Alzheimer’s disease, in combination with the aminoglycoside antibiotic, amikacin (AMK), to treat *P. aeruginosa* and CRPA (carbapenem-resistant *P. aeruginosa*) infections in vitro and in vivo. The researchers found that MEM indirectly promoted ROS generation in neutrophils by increasing expression of the p67phox subunit of NADPH oxidase, which enhanced the bactericidal effect of neutrophils against *P. aeruginosa*. Further, MEM was found to be synergistic with AMK, and in vivo experiments showed that the combination therapy reduced the severities of bacteremia and inflammation and that the bacterial burden load was reduced in rats [190].

Host-directed therapies offer the benefit of targeting the host, which greatly reduces the risk of inducing drug resistance in the bacteria. However, the infection environment is complicated, and monotherapies could become ineffective against infections where the bacteria are highly adaptable and have the ability to survive intracellularly, such as what we have described here for the non-canonical intracellular pathogens *S. aureus* and *P. aeruginosa*. Several recent encouraging studies have been published on host-directed monotherapies, including: using the kinase inhibitor Ibrutinib to treat intracellular *S. aureus* [191]; using the sarco/endoplasmic reticulum Ca^2+^-ATPases (SERCA) inhibitor thapsigargin to treat intracellular *S. aureus* [192]; treating with the pan-caspase inhibitor quinoline–valine–aspartic acid–difluorophenoxymethyl ketone (Q-VD-OPH) to effectively reduce bacterial skin infections caused by *S. aureus*, *P. aeruginosa*, and *Streptococcus pyogenes* [193]; and utilizing liposomes loaded with phosphatidylinositol 5-phosphate (PI5P), which is involved in actin regulation and vesicular trafficking, to effectively aid macrophage killing of *P. aeruginosa* in a CF model [194]. These host-directed monotherapies offer many possible avenues for treating difficult, persistent bacterial infections, and when these are combined with traditional antibiotic therapies, the outcomes could be enhanced. Ultimately, to combat the growing number of AMR infections worldwide, a multipronged therapy will be required. Thus, there continues to be an urgent need for new therapies to combat AMR infections, and research into combination therapies, including ones that include host-directed therapies, should be a priority to accelerate treatment while slowing the spread of AMR.

## 5. Surveillance and Prevention Are Instrumental in Fighting the AMR Crisis

### 5.1. Overview

Recognizing the full life cycle and niche capabilities in infection caused by *P. aeruginosa* and *S. aureus* is critical for proper treatment and for reducing the spread of AMR. Additionally, global surveillance and prevention options are essential not only for controlling the infections caused by these two specific priority ESKAPE pathogens but also for controlling the broad spread of AMR in general.

### 5.2. Surveillance Is the Cornerstone of AMR Mitigation

Surveillance lays the groundwork for the control of AMR spread. The WHO Global Antimicrobial Resistance Surveillance System (GLASS) AMR surveillance initiative was launched in 2015 to standardize global reporting of AMR data. The fourth GLASS report published in 2021 [3] included AMR data on over 3 million laboratory-confirmed infections reported in 70 countries. Alarmingly, it was shown that high rates of resistance to both first-line drugs (i.e., co-trimoxazole) and last resort antibiotics (i.e., carbapenems) was observed globally. While it is worth noting that AMR can happen naturally in microbes as a spontaneous phenomenon, the misuse and/or overuse of antimicrobial drugs is the key driver for rapid development and spreading of AMR [195]. An important new development that was highlighted in this 2021 GLASS report was the antimicrobial consumption (AMC) surveillance at the national level. Another key finding from the AMR crisis in the GLASS report was that antimicrobial use in humans continues to increase. Geographical coverage of the GLASS initiative has been continuously expanding: as of May 2021, 109 countries and territories worldwide have enrolled in GLASS [3]. Even as the inaugural issue containing the AMC surveillance data, the 2021 GLASS report included the data reported from 15 out of 19 countries that are enrolled in the AMC module. Nevertheless, broader participation involving countries with different levels of incomes from all regions will facilitate the WHO’s mandate of coordinating international efforts to mitigate the impact of AMR. For example, India, China, and the USA had the highest antibiotic consumption rate across countries in 2015 according to a report analyzing global antibiotic consumption [196]. In China, two national antimicrobial resistance surveillance networks were established in 2005: The China Antimicrobial Resistance Surveillance System and the China Antimicrobial Surveillance Network publish their summary reports annually [197,198]. Despite some gaps in reporting methodologies in these reports, recent efforts have been undertaken to standardize surveillance on the national level in China [199,200]. The inclusion of these data as an integral part of the GLASS initiative will facilitate the concerted actions for global AMR surveillance.

### 5.3. The Use of Vaccines Is Critical for Limiting Antimicrobial Use, and Thereby AMR Development

Limiting the use of antimicrobial drugs is another key component of AMR control. Vaccines for both humans and animals are instrumental in preventing infections and reducing antimicrobial use, therefore limiting the development of resistance. The use of vaccines to fight against infectious diseases is the most significant advance in medical history. As reviewed previously [201], vaccines against viral infections have been widely used (i.e., for measles and influenza) and have gained tremendous success, including the eradication of smallpox and the recently developed revolutionary mRNA vaccines against SARS-CoV-2 [202,203]. Similarly, vaccines targeting bacterial infections have saved countless lives from preventable diseases, such as diphtheria, tetanus, tuberculosis, meningitis, and pneumonia. Table 5 summarizes currently licensed vaccines against bacterial pathogens, which belong to four major different types: inactivated, attenuated, toxoid, and subunit/conjugated. Some of the early vaccines against typhoid fever [204] and cholera [205] belong to the inactivated category, which uses heat- and chemical-killed whole bacterial cells as the main antigen composition. The only inactivated vaccines that are in common usage today are some of the ones used for treating *Bordetella pertussis*-caused whooping cough [206], while the others are for specific regional use only. For example, the currently licensed inactivated oral cholera vaccines are mostly used within disease endemic regions and for travelers to those regions [207]. The attenuated category uses live whole bacterial cells with abrogated virulence as the vaccine strains. For example, the vaccine strain Bacille Calmette-Guérin (BCG) is a modified *Mycobacterium bovis* that is still widely used in some regions [208] to elicit a protective response against the closely related *Mycobacterium tuberculosis*, the causative agent of tuberculosis. Similarly, *Salmonella* Typhi strain Ty2 [209,210] and *Yersinia pestis* strain EV76 [211] were used as attenuated vaccine strains for typhoid and plague, respectively. With better understanding of molecular pathogenesis of various pathogens, this vaccination strategy was also exploited for other enteric pathogens, although they were either discontinued (i.e., for *Vibrio* [212]) or are still under development (i.e., *Shigella* and enterotoxigenic *Escherichia coli* [213,214]). Other major breakthroughs in vaccine development enabled by our better understanding of infection and immunity mechanisms include toxoid and subunit/conjugated vaccines. The toxoid vaccines, including diphtheria or tetanus toxoid vaccines that are broadly used worldwide, utilize inactivated toxins as antigens when specific toxins instead of the microorganisms cause diseases [215]. The subunit vaccines (also known as acellular vaccines) contain purified fragments from a pathogen, such as proteins or polysaccharides that specifically elicit protective immunity. Examples include vaccines for pertussis (protein subunit), meningitis, and pneumonia (polysaccharide). Some surface polysaccharides can also be fused with a carrier protein in conjugated vaccines to elicit a T cell-dependent longer lasting immunity [216,217]. Despite the tremendous progress in the use of vaccines to prevent bacterial infections, we still lack licensed vaccines for important bacterial pathogens. Examples include the ones that belong to the WHO’s AMR priority species, such as methicillin-resistant *Staphylococcus aureus* [218] and carbapenem-resistant Gram-negative *Pseudomonas aeruginosa* [219], *Acinetobacter baumannii*, *Klebsiella pneumoniae*, and *E. coli* [220]. Moreover, pathogens that cause sexually transmitted diseases (i.e., *Treponema pallidum*, *Chlamydia*, and *Neisseria gonorrhoeae*) [221,222] are also developing antibiotic resistance. Meanwhile, although many veterinary vaccines for livestock are used to control parasitic diseases [223], no licensed vaccines against parasitic or fungal infections are available for human use, despite the large burden of both infection types on global health systems and antimicrobial consumption [224,225].

## 6. Concluding Remarks

Excessive use of antibiotics has accelerated the emergence of multidrug-resistant and extensively drug-resistant bacteria, against which even the most effective drugs are ineffective. The WHO has been sounding the alarm about antimicrobial resistance (AMR) as a severe threat to global health and the global economy [3,9,226]. Existing models predict that AMR-associated infections could cause naturally occurring global infectious catastrophes and drive millions of people into extreme poverty. AMR is a highly complex issue, therefore requiring interdisciplinary and multisectoral efforts to better understand and tackle this problem. Better understanding of resistance mechanisms can facilitate the surveillance of AMR emergence and epidemiology and the development of vaccines to prevent and new treatments to treat infections, which are all critical in our battle with the global AMR crisis.

Among the list of these AMR-associated bacteria, carbapenem-resistant *P. aeruginosa* along with methicillin- and vancomycin-resistant *S. aureus* cause notoriously difficult-to-treat recalcitrant infections that are associated with the highest risk of mortality and increased health care costs [227]. Neither of these two species is considered a canonical intracellular bacterium. However, emerging evidence has shown that both *P. aeruginosa* and *S. aureus* exploit the host intracellular environment actively by the use of multiple virulence factors to promote their intracellular life cycles, potentially contributing to their survival in the presence of antibiotics. This poses an additional challenge to eradicate infections caused by these bacteria, which are already highly resistant to antibiotics. Further research into the mechanisms driving these two bacterial species’ intracellular life cycles are desperately needed. Additionally, research in developing novel therapeutics and treatment strategies are urgently needed. In our review, we did not try to exhaustively cover all novel therapies, such as all combination therapies, phage therapies, etc. Instead, we focused on highlighting recent research on therapies that harness the host’s immune response, since it is more difficult for the bacteria to develop resistance to the treatment because it is host-directed. Given the complexity of these intracellular infections, we believe a multifaceted treatment approach will be required to avert catastrophes caused by the increasing number of AMR infections worldwide. Using the host’s immune response to assist in effectively eradicating the intracellular pathogen will be a key component in slowing the spread of AMR infections, and combining these treatments with traditional antimicrobials has the potential to accelerate patient care and recovery.

## Figures and Tables

**Table 1:** Common classes of antibiotics in use, their mode of action, and host cell permeability.

Antibiotic Class	Mode of Action	Host Cell Permeable [25,26,27]
Aminoglycosides	Inhibit protein synthesis	Yes, some antibiotics in this class enter host cells via endocytosis
Ansamycins	Inhibit RNA synthesis	Yes, rifamycin enters via passive uptake (diffusion)
β-Lactams	Inhibit cell wall synthesis	Yes, small molecules via diffusion, larger molecules possibly via endocytosis
Chloramphenicol	Inhibits protein synthesis	No, requires modification for enhanced entry into host cells
Glycopeptides	Inhibit cell wall synthesis	No, requires modification for enhanced entry into host cells
Lipopeptides	Disrupt cell membrane functions	No/Unknown
Macrolides	Inhibit protein synthesis	Yes, diffusion and partly active uptake
Oxazolidinones	Inhibit protein synthesis	Yes, passive uptake
Quinolones	Interfere with bacterial DNA replication	Yes, active and passive cellular uptake, depending on the quinolone
Streptogramins	Inhibit protein synthesis	No/Unknown
Sulfonamides	Inhibit folic acid synthesis	Yes, active uptake
Tetracyclines	Inhibit protein synthesis	Yes, active uptake

**Table 2:** List of *P. aeruginosa* strains and their expression of ExoS or ExoU.

Strain	ExoS	ExoU
PAO1	+	−
CF18	+	−
CF27	+	−
PAK	+	−
JJ692	−	+
E2	+	−
MSH10	+	−
X13273	−	+

**Table 3:** Alternative staphylococcal secondary mechanisms for cellular attachment and potential entry.

*S. aureus* Component	Host Component	Bridge	Host Cell Type	References
Atl	Heat shock cognate protein 70		Keratinocytes	[87]
			Endothelial cells	[88]
ClfA	αvβ3 integrins	Fibrinogen	None reported	[89]
			Vascular endothelial cells	[90]
	Annexin A2		MAC-T cell	[91]
	Von Willebrand Factor	Von Willebrand binding protein	Endothelial cells	[92,93,94]
ClfB	Plasma fibrinogen			[95]
	Cytokeratin 10		Desquamated epithelial cells	[96]
	Loricrin		Squamous epithelial cells	[97,98]
IsdB	β3-containing integrins	Extracellular matrix Vitronectin	HEK-293T, HeLa	[99]
	αvβ3 integrins		Epithelial/endothelial cells	[100]
Lpl	Hsp90		Keratinocytes	[101,102]
SraP	gp340		A549 cells	[103]
SdrD	Desmoglein 1		Keratinocytes	[104]
			Desquamated nasal cells	[105]

**Table 4:** Prevalence of genes encoding primary and secondary staphylococcal mechanisms of cellular entry in *S. aureus* isolates genome.

*S. aureus* Component	Gene Prevalence in the Investigated *S. aureus* Isolates’ Genome	References
Atl	100%	[119,120,121,122]
ClfA	100%	[118,119]
	87%	[120]
	82%	[121]
	70.4%	[123]
ClfB	100%	[119,121]
	98%	[120]
Eap	100%	[119,124]
	99%	[120]
	45%	[121]
FnBPA	100%	[119,120]
	99%	[121]
FnBPB	100%	[119]
	73%	[121]
	44%	[120]
HLA	100%	[120,121,125]
	96%	[117]
	91.9%	[123]
	90.3%	[118]
IsdB	97%	[121]
	94%	[120]
SpA	100%	[120,121]
	90%	[117]
SraP	100%	[121]
	43%	[120]
SdrD	91%	[121]
	40%	[119]
	36%	[120]

**Table 5:** Different types of bacteria vaccines approved for use.

Disease(s)	Bacterial Pathogen	Vaccine Type
diphtheria	*Corynebacterium diphtheriae*	toxoid
tetanus	*Clostridium tetani*	toxoid
whooping cough	*Bordetella pertussis*	subunit or inactivated
meningitis/pneumonia	*Haemophilus influenzae*	conjugated
meningitis/pneumonia	*Streptococcus pneumoniae*	subunit or conjugated
meningitis	*Neisseria meningitidis*	subunit or conjugated
typhoid fever	*Salmonella typhi*	attenuated or subunit
cholera	*Vibrio cholerae*	inactivated
plague	*Yersinia pestis*	inactivated
anthrax	*Bacillus anthracis*	subunit
tuberculosis	*Mycobacterium tuberculosis*	attenuated
tularemia	*Francisella tularensis*	attenuated
typhus	*Rickettsia prowazekii*	inactivated
Q fever	*Coxiella burnetii*	inactivated

## Data Availability

Not applicable.
